# Corrosion Resistance of Concrete Reinforced by Zinc Phosphate Pretreated Steel Fiber in the Presence of Chloride Ions

**DOI:** 10.3390/ma13163636

**Published:** 2020-08-17

**Authors:** Xingke Zhao, Runqing Liu, Wenhan Qi, Yuanquan Yang

**Affiliations:** 1 School of Materials Science and Engineering, Shenyang Ligong University, Shenyang 110159, China; zczczc2000@126.com (X.Z.); aquarius0109@163.com (Y.Y.); 2 Technology R & D Department, Zhejiang Dadongwu Group Co., Ltd., Huzhou 313000, China; an77qt@163.com

**Keywords:** steel fiber reinforced concrete, zinc phosphate, chloride ion corrosion resistance, microstructure

## Abstract

This paper aims to provide new insight into a method to improve the chloride ion corrosion resistance of steel fiber reinforced concrete. The steel fiber was pretreated by zinc phosphate before the preparation of the fiber reinforced concrete. Interfacial bond strength, micro-hardness and micro-morphology properties were respectively analyzed in the steel fiber reinforced concrete before and after the chloride corrosion cycle test. The results show that the chloride ion corrosion resistance of the steel fiber was enhanced by zinc phosphate treatment. Compared to plain steel fiber reinforced concrete under chloride ion corrosion, the interfacial bond strength of the concrete prepared by steel fiber with phosphating treatment increased by 15.4%. The thickness of the interface layer between the pretreated steel fiber and cement matrix was reduced by 50%. The micro-hardness of the weakest point in the interface area increased by 54.2%. The micro-morphology of the interface area was almost unchanged before and after the corrosion. The steel fiber reinforced concrete modified by zinc phosphate can not only maintain the stability of the microstructure when corroded by chloride ion but also presents good bearing capacity.

## 1. Introduction

Steel fiber reinforced concrete (SFRC) is a new type of multiphase composite material which is formed by mixing short steel fibers with random distribution in plain concrete [1,2,3]. For concrete, steel fiber can prevent cracks and increase the toughness of the concrete [4,5]. However, chloride ion, as a corrosive medium in the marine environment, can easily cause corrosion of steel fiber, which leads to loss of bearing capacity and toughness of steel fiber reinforced concrete members [5,6,7]. In the process of preparation of steel fiber reinforced concrete, a layer of interface phase with a thickness of around 50–100 μm will be formed when the steel fiber is wrapped by the substrate. Due to the particularity of its structure, the bond strength, fracture toughness, and hardness of the interface are all weak points in SFRC [8]. Unfortunately, the corrosion of steel fiber under chloride ion attack will seriously affect the performance of the interface area, resulting in the reduction of the performance of steel fiber reinforced concrete. Therefore, the corrosion protection of steel fiber reinforced concrete should not be ignored [9,10,11,12,13,14].

There are many factors affecting the corrosion of steel fiber in concrete, such as surrounding environment, selection of material as ingredients for concrete [15], steel fiber condition, exposure time, and cracks resulting from the brittle nature of the concrete [16,17]. Thus, careful selection of materials, suitable mixing proportion design for concrete, and protection for the steel fiber are beneficial for the anti-corrosion of steel fiber. Previous studies [18] have found that a high water/binder ratio for concrete under a corrosive environment resulted in accelerated corrosion of steel fiber. Concrete with low water/binder ratio and permeability can resist chloride penetration into the concrete matrix and provide a barrier against oxygen entry and, therefore, the time of corrosion initiation for the steel fiber can be extended. In addition, waste by-products like fly ash, silica fume, and granulated blast furnace slag were used to partially replace the binder, which decreased the permeability of the concrete and thus the steel fiber was protected from corrosion [19,20,21]. Research works also concentrate on methods to increase the crack resistance of concrete structures and therefore lessen the attacks of aggressive agents such as chloride ions, including the addition of steel lining and/or pre-stressing of the concrete. Moreover, cracks of the concrete are repaired by epoxy resins and/or urethanes [5]. All these methods have their limitations. Careful selection of materials for concrete and suitable mixing design mostly work on dormant cracks and thus the steel fibers are protected, but when the concrete is continuously subjected to dynamic cyclic loads, as in an offshore environment, it fails to protect the steel fiber. The additional steel lining and pre-stressing for the concrete significantly increase the construction cost as well. Moreover, for steel fiber reinforced concrete, the fibers are dispersed in the entire matrix, located close to the surface and with less concrete matrix cover. Therefore, corrosion of steel fiber cannot be avoided and effective methods are required in order to solve this problem.

Recently, researchers found that phosphating was an effective and important process applied to steels which has been successfully used in automotive industries due to easy operation and low cost [22]. The phosphating method (phosphate coats) can improve the corrosion resistance and lubrication properties of steel fibers [23] as well as steel rebars [24,25]. Among phosphate coats, zinc phosphate is the most popular coating, due to its capacity for anodic protection of Zn^2+^ and barrier protection of the existing phosphate film, and, therefore, phosphate coatings have attracted more attention in recent years. Phosphating as an established method is used in various industries and a majority of the phosphating is applied in automotive industries. However, zinc phosphating of steel fiber for concrete has not been sufficiently investigated. The strength of the interfacial bond between the zinc phosphate treated steel fiber and the concrete matrix and the ways in which microstructures of the concrete matrix are affected by the incorporation of the pretreated steel fiber are not clear. These are the key parameters that affect the durability of the concrete [26].

This work thus aims to investigate the interfacial bond strength and microstructure of steel fiber reinforced concrete by zinc phosphate treatment on the steel fiber. The steel fiber was pretreated by zinc phosphate before the preparation of the fiber reinforced concrete. Interfacial bond strength, micro-hardness, and micro-morphology properties were respectively analyzed in the prepared steel fiber reinforced concrete before and after the chloride corrosion cycle test. The concrete with modified steel fiber could be more suitable to be the first choice material of load-bearing structures in severe corrosive environments so as to greatly expand the scope of use of steel fiber reinforced concrete. It can provide safer and more reliable load-bearing materials for future engineering construction in various corrosive environments.

## 2. Experimental Methods

### 2.1. Materials

CEM 42.5 Portland cement produced by Ji Dong Cement Co., Ltd. of Anshan, Liaoning, China, was used in the experiment. Grade II fly ash was produced by Shenhai Thermal Power Plant, Shenyang, China. The chemical composition and physical properties of Portland cement and fly ash are presented in Table 1. The steel fiber produced by Shanghai Shiweike Industry (Shanghai, China) and Trade Co., Ltd. (Shanghai, China) has a fiber length of 40 mm, a fiber length diameter ratio of 69.3, and a tensile strength of ≥600 MPa. River sand (medium sand) with fineness modulus of 2.60 and crushed stone with particle size of 5–25 mm were also used when making the concrete specimens.

### 2.2. Methods

**Figure 1:**
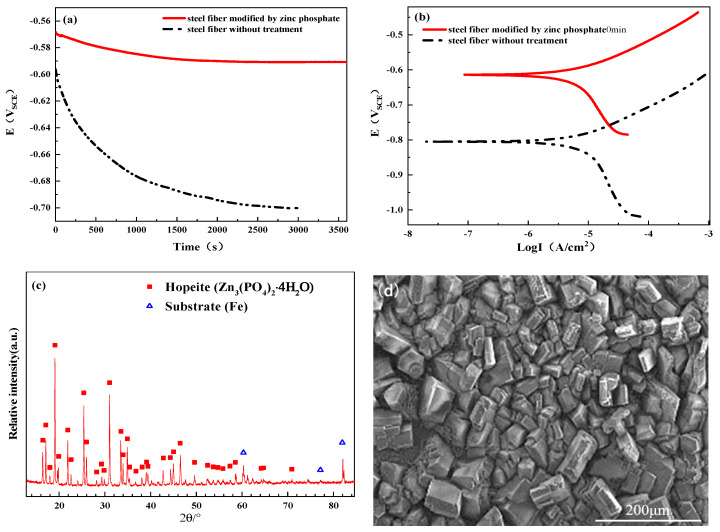
Performance index of the steel fiber modified by zinc phosphate at 75 °C for 20 min: (**a**) ppen circuit voltage, (**b**) polarization curve, (**c**) XRD identification, and (**d**) SEM morphology.

Phosphating solution preparation: 8.0 g ZnO powder and moderate amounts (about 15 g) of distilled water were mixed; then, 20 mL of 85% H_3_PO_4_ was added as well as 1.0 g of citric acid, 0.2 g of Ca(NO_3_)_2_, and 0.2 g of Zn(NO_3_)_2_. The composition of this mixture after reactants’ reaction mainly comprised of Zn_3_(PO_4_)_2_ and minor amounts of Ca(NO_3_)_2_ and Zn(NO_3_)_2_, which could also be seen from the XRD identification (Figure 1c) of the coating of the steel fiber that was modified by the as-prepared zinc phosphate solution. The addition of minor amounts of Ca(NO_3_)_2_ and Zn(NO_3_)_2_ here was to improve the formation of the zinc phosphate coating layer for the steel fiber. Finally, additional distilled water was added in order to obtain 1 L of the phosphating solution.

During the treatment of steel fiber by zinc phosphate solution, the oil and rust on the steel fiber was firstly removed, and it was then treated with the zinc phosphate solution. Each process was performed separately and in turn. In particular, in the process regarding the oil and rust removal from the steel fiber, the steel fiber was treated in accordance with the following procedures: (a) polished, (b) soaked in 10% NaOH solution at 50 °C in water-bath for 10 min, (c) soaked in acetone for 2 min and then distilled water for another 2 min before being dried, (d) soaked in 25% H_2_SO_4_ solution at 60 °C in water bath for 5 min, (e) repeat the third step, (f) soaked in 25% H_3_PO_4_ at ambient temperature for 5 min, and (g) soaked in 6% citric acid solution for 2 min before being dried. According to the results of previous experiments, the steel fiber was phosphatized at 75 °C for 20 min to obtain better performance. The performance index of the steel fiber modified by zinc phosphate at 75 °C for 20 min is shown in Figure 1. The open circuit voltage and polarization curve (Figure 1a,b) indicates that steel fiber modified by zinc phosphate presents better corrosion resistance than the non-treated one. Moreover, the coated layer of the steel fiber modified by zinc phosphate mainly contains a hopeite phase ((Zn_3_(PO_4_)_2_·4H_2_O), Figure 1c) with irregular structures (Figure 1d), as well as an Fe phase (substrate).

Electrochemical tests were performed on the plain steel fiber and zinc phosphate modified steel fiber. During the electrochemical test, the samples were suspended in 3.5 wt.% NaCl solution at a scan rate of 0.01 mV/s from −0.2~0.67 V using an EG and G237 electrochemical workstation. The corrosion potential (E_corr_), corrosion currency density (I_corr_), polarization resistance (R_p_), corrosion rate, porosity of the coating layer (P), and corrosion protection efficiency (P_e_) for the prepared steel fiber phosphatized at 75 °C for 20 min and that of plain steel fiber were determined by the Tafel extrapolation method [27] and are shown in Table 2.

Concrete specimens with a water to binder ratio of 0.46 and 1% volume of steel fiber (in relation to the total volume of concrete) were prepared according to the “Test method for steel fiber reinforced concrete (CECS 1389, China standard)”. The detailed mixing proportions are presented in Table 3.

The concrete specimens with sizes of 100 mm × 100 mm × 400 mm were treated 30 times with chloride ion solution cycles. For each cycle, the specimens were immersed in 6% NaCl solution for 12 h at 20 °C and then dried for another 12 h. After the chloride ion corrosion test, the concrete specimens were tested for various tests. Microstructures of the corresponding specimens were observed by SEM (S-4800, Hitachi, Tokyo, Japan).

Crushed concrete specimens (5 mm in diameter) that contained interfacial transition zones between the steel fiber and cement matrix were chosen for micro-hardness measurement (FM-700 micro-hardness tester, Tokyo, Japan, FUTURE-TECH). Each sample was polished to obtain a smooth fracture surface before the micro-hardness measurement, to ensure its accuracy. Micro-hardness data were recorded from the substrate of the cement matrix at various points around 100 μm away.

The interfacial bond strength between the steel fiber and the cement substrate was measured according to the “Test method for steel fiber reinforced concrete (CECS 1389, China standard)”. For the micro-hardness and interfacial bond strength tests, three samples (with the same type of steel fiber and under the same environment) were tested.

## 3. Results and Discussion

### 3.1. Bond Strength

**Figure 2:**
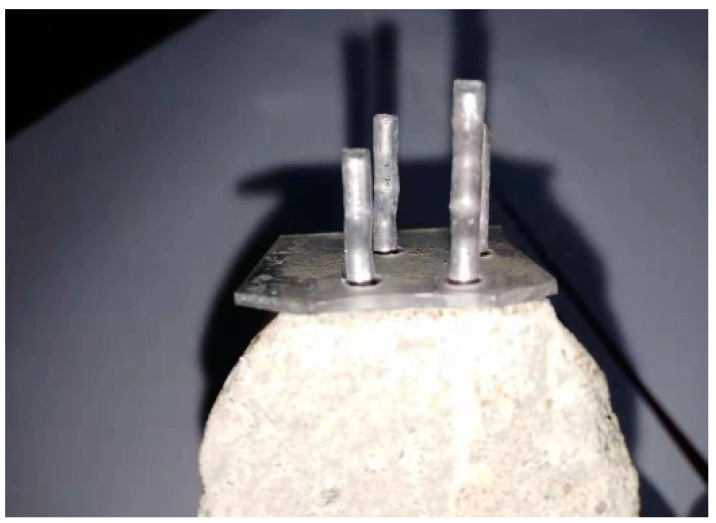
Pull-out test for the steel fiber from the cement matrix.

Two forces exist in the process of pulling out steel fiber from the cement matrix. Cohesive force plays a key role between the fiber and cement matrix before the arrival of a peak load, and when the fiber de-bonds from the cement matrix, friction between the fiber and the cement matrix becomes the main working factor. A diagram showing the steel fiber pull-out from the cement matrix is shown in Figure 2.

Generally, the load displacement curve produced by fiber pull-out is divided into three stages. In the first stage, the interface between the steel fiber and concrete is elastic, which is referred to as linear loading. In the second stage, the steel fiber slips from the concrete and gradually loses its stability, which is referred to as non-linear loading. In the third stage, the steel fiber is de-bonded from the concrete matrix, and the steel fiber is pulled out of the concrete matrix, which is referred to as non-linear unloading. The mathematical expression of bond strength between steel fiber and concrete matrix is given in Equation (1).

(1) $$f_{fu}=\frac{F_{fu}}{nu_{f}l_{fe}}$$

where *f_fu_* denotes the interfacial bond strength of steel fiber and concrete (MPa), *F_fu_* is the maximum load when steel fiber is pulled out (N), *n* is the embedded quantity of steel fiber, *u_f_* is the perimeter of the steel fiber cross-section (mm), and *l_fe_* is the embedded length of the steel fiber (mm).

The toughening effect of steel fiber on concrete is mainly due to the large amount of work required when the fiber is pulled out from the matrix. Therefore, the bond strength between the steel fiber and matrix determines its toughening effect on the concrete. The mechanical properties of the steel fiber in the test were obtained by calculation, as presented in Table 4.

Table 4 shows that the corrosion environment has a great influence on the interfacial bond performance of plain steel fiber (without treatment) reinforced concrete. The average bond strength of the interface between steel fiber and concrete obviously decreased after corrosion, as well as the work carried out when the fiber was pulled out. The average bond strength of the interface between the steel fiber and cement was 15.4% lower than that of phosphatized steel fiber, and 15.0% less work was required when the fiber was pulled out. It can be seen that the corrosion resistance of steel fiber has been greatly improved after phosphating treatment. This can ensure improvement in the corrosion resistance and bond strength of the interface after initiation of corrosion.

**Figure 3:**
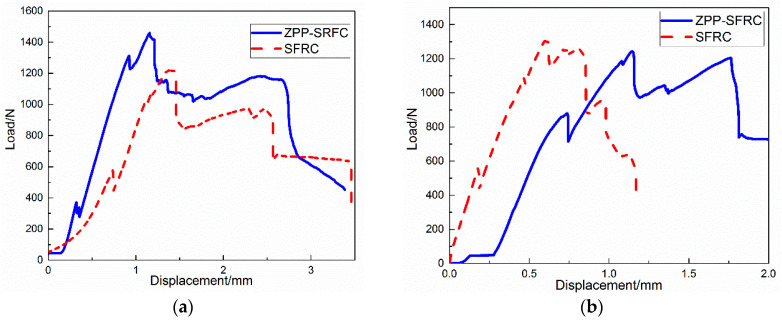
The relationship between load and displacement of the samples under different environments in the pull-out test: (**a**) NaCl solution and (**b**) without corrosion.

To further study the influence of phosphatized steel fiber on the interfacial bond strength of concrete specimens under different corrosion environments, the relationship between the load and displacement of the steel fiber reinforced concrete specimens in different corrosion environments before and after phosphating was plotted by recording and taking the average value of each type of data (Figure 3).

By comparing the load displacement curve of steel fiber reinforced concrete modified by zinc phosphate and plain steel fiber reinforced concrete, it can be seen that the ultimate load strength of steel fiber reinforced concrete modified by zinc phosphate was significantly higher than that of plain steel fiber reinforced concrete in a chloride corrosion environment. The difference in ultimate load between steel fiber reinforced concrete modified by zinc phosphate and plain steel fiber reinforced concrete was not significant in the environment without corrosion. The displacement of steel fiber reinforced concrete modified by zinc phosphate, however, shifted around 0.6 mm towards the right compared to plain steel fiber reinforced concrete, as well as withstanding higher load. This demonstrates that the steel fiber modified by zinc phosphate has good interfacial adhesion.

### 3.2. Micro-Hardness

**Figure 4:**
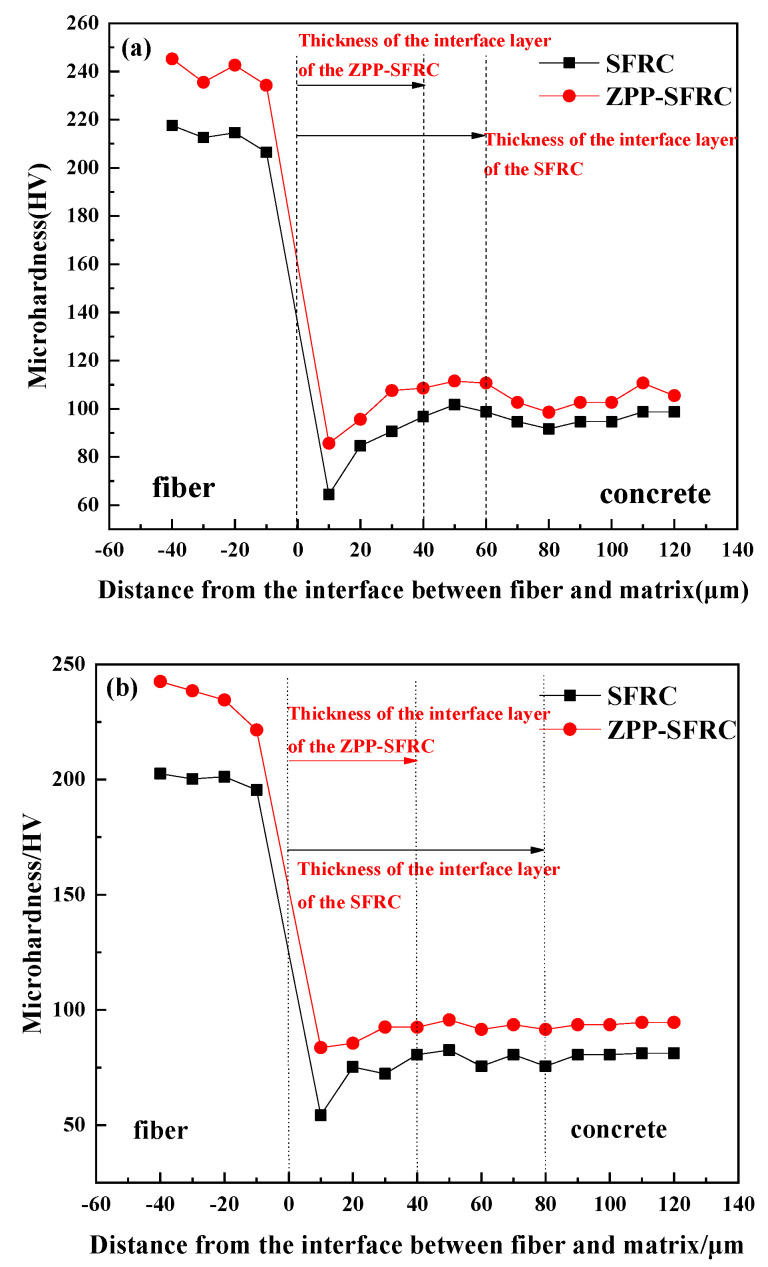
Micro-hardness of the interface layer between ZPP-SFRC (zinc phosphate modified steel fiber reinforced concrete) and SFRC (steel fiber reinforced concrete) under different environments: (**a**) without corrosion and (**b**) with NaCl corrosion.

**Figure 5:**
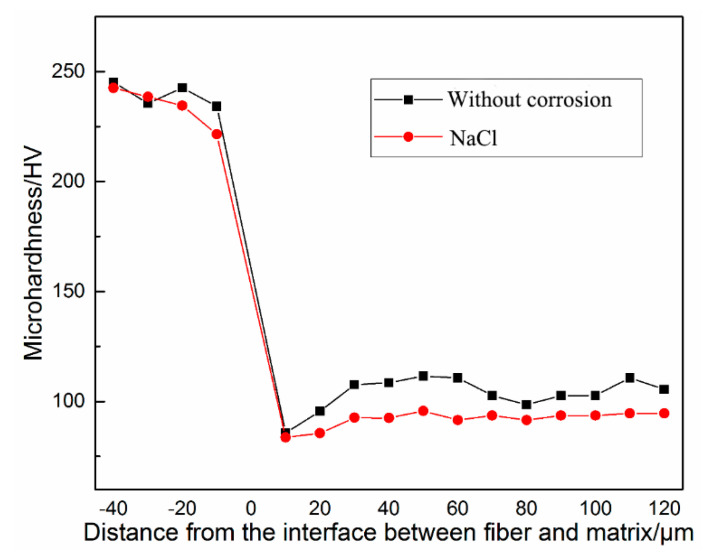
Micro-hardness of interface layer between zinc phosphate modified fiber and concrete matrix.

For steel fiber concrete, the interface layer is a contact layer between the fibers and the concrete matrix. The micro-hardness value of the interface layer is an intuitive response to the performance of the interface area. It can reflect the interface effect between the steel fiber and concrete matrix and the size of the interface area. The sample corroded by NaCl solution was polished and leveled and then observed by a micro-hardness tester. The micro-hardness values from the tests are presented in Figure 4. The comparison of the interface between steel fiber and concrete modified by zinc phosphate in a chloride corrosion environment and non-corrosion environment is shown in Figure 5.

It can be seen from Figure 4 that phosphating treatment decreases the thickness of the interface layer between the steel fiber and concrete by 20 μm, as well as increasing its micro-hardness by around 20 HV in both corroded and uncorroded environments. With regards to the plain steel fiber reinforced concrete, the presence of a corroded environment increases the thickness of the interface layer by 20 μm and decreases the micro-hardness by 15 HV, compared to that under the uncorroded environment. However, the thickness of the interface layer of the steel fiber reinforced concrete modified by zinc phosphate did not change much and remained at around 40 μm. In the process of steel fiber extending to the concrete matrix, the micro-hardness value decreased initially and then increased and finally tended to be stable. There was a minimum value of micro-hardness curve, and the weak valley was the most vulnerable part of the structure. Compared with the micro-hardness value of the weakest point of the interface, the micro-hardness of the weakest point of the sample increased, regardless of whether it was corroded by NaCl solution or not after phosphating, especially for the sample after corrosion. The micro-hardness value of the weakest point of the interface of plain steel fiber reinforced concrete after NaCl corrosion was 54.257 MPa, while this value was 83.654 MPa for the steel fiber reinforced concrete modified by zinc phosphate, which increased by 54.2%. The results show that the interface structure between the steel fiber and concrete matrix can be effectively improved by zinc phosphate modification. It can decrease the thickness of the interface layer, improve the hardness of the interface layer, reduce the influence of chloride corrosion on the interface layer of steel fiber reinforced concrete, and finally improve the interface performance.

It was found that the micro-hardness of the interface between the steel fiber reinforced concrete modified by zinc phosphate and that corroded by NaCl solution are similar. The change in the range of micro-hardness was around 1%. This shows that the corrosion of the NaCl solution has little effect on the interface layer between the steel fiber and concrete matrix modified by zinc phosphate, which is due to the good corrosion resistance of the zinc phosphate layer, which can resist chloride corrosion and bond well with the concrete matrix.

### 3.3. SEM-EDS Analysis

**Figure 6:**
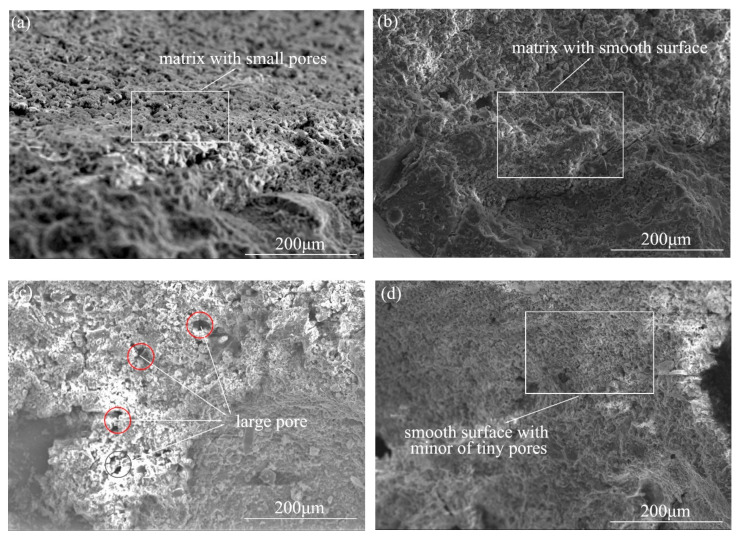
Microstructures of the matrix from SFRC and ZPP-SFRC before and after corrosion: (**a**,**b**) before corrosion, (**c**,**d**) after corrosion.

The microstructure of the concrete matrix after the pull-out of the fiber is presented in Figure 6. The interface area adjacent to the steel fiber for the SFRC (Figure 6a) was slightly different from that of ZPP-SFRC (Figure 6b), which tends to be a porous structure with small pores rather than a simple, dense, and smooth structure with calcium silicate hydrate (C–S–H) gel. This could be attributed to the different properties of the plain steel fiber and zinc phosphate modified fiber in the vicinity of their surfaces. In the process of hydration, the cement hydrates well as a result of the fact that more water was absorbed by the zinc phosphate modified fiber in the vicinity of their surfaces, thus forming a denser and smoother structure (Figure 6b). After NaCl corrosion, the structure of the steel fiber concrete without phosphating treatment has quite a high number of large pores (Figure 6c). However, the micro-morphology of steel fiber reinforced concrete modified by zinc phosphate did not change much and only a low quantity of small pores appeared (Figure 6d). This may be due to the excellent corrosion resistance and surface roughness of zinc phosphate modified steel fiber. The excellent corrosion resistance of the zinc phosphate modified steel fiber could prevent the growth of rust crystals on its surface, which could mitigate the expansion by the rust crystal formation; thus, the cracks on the concrete proceed very slowly and the corrosion resistance of the steel fiber concrete is improved.

**Figure 7:**
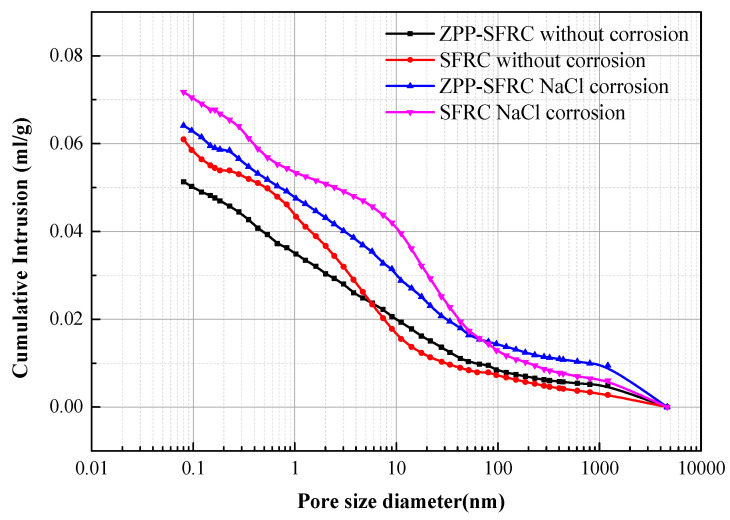
Mercury intrusion of ZPP-SFRC and SFRC.

A mercury pressure intrusion test can be used to analyze the pore structure of concrete. Figure 7 is a comparison of mercury intrusion before and after NaCl solution corrosion between zinc phosphate modified steel fiber concrete and plain steel fiber concrete. After NaCl corrosion, the cumulative mercury intrusion of steel fiber reinforced concrete is higher than that of zinc phosphate modified steel fiber. It shows that the porosity of steel fiber reinforced concrete is higher than that of zinc phosphate modified steel fiber.

**Figure 8:**
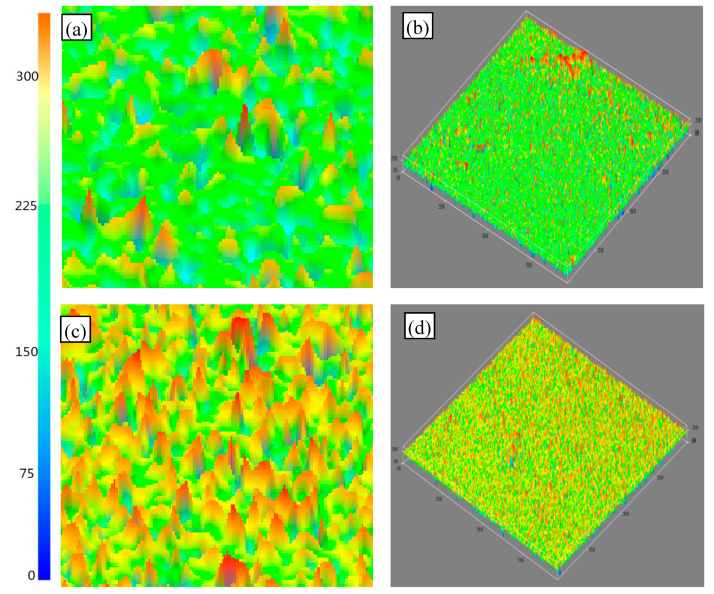
Morphology of plain steel fiber and zinc phosphate modified steel fiber: (**a**) 2D morphology of plain steel fiber, (**b**) 3D morphology of plain steel fiber, (**c**) 2D morphology of phosphatized steel fiber, and (**d**) 3D morphology of zinc phosphate modified steel fiber.

The three-dimensional and plane morphology of the steel fiber that pulled out from the concrete was observed by Atomic Force Microscope (AFM, Figure 8). The surface of plain steel fiber is relatively flat, with little fluctuation, as compared to zinc phosphate modified steel fiber. However, the overall thickness of zinc phosphate modified steel fiber is relatively higher, the surface undulation has an obviously cone-shaped structure, and the particles are closely arranged due to the existence of the zinc phosphate coating.

**Figure 9:**
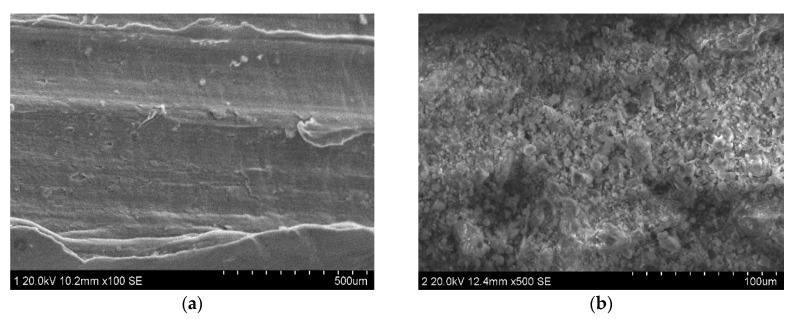

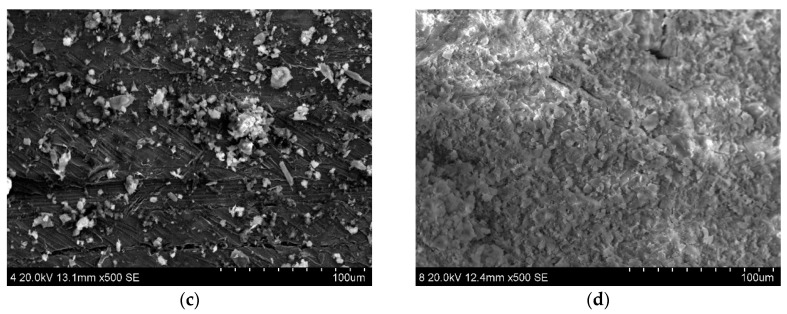
Micrograph of steel fiber pulled out from the concrete: (**a**) before corrosion, plain steel fiber; (**b**) before corrosion, zinc phosphate modified steel fiber; (**c**) after corrosion, plain steel fiber; (**d**) after corrosion, zinc phosphate modified steel fiber.

Figure 9 shows the micrographs of plain steel fiber and zinc phosphate modified steel fiber taken out from the crushed concrete before and after corrosion. The internal pH of concrete was around 12.5–13.5 in the alkaline environment, in which steel fiber forms a passive film on its surface to protect itself. The micrograph of the steel fiber from the concrete without corrosion (Figure 9a) indicated that the surface of the steel fiber was relatively clean and there was almost no cement hydration product attached to it. The content of Ca was only 5.53% and that of Fe was 84.14%, indicating that the bond strength between steel fiber and concrete matrix was weak. There was almost no cohesiveness between hydration products and steel fiber, where the bond strength of the interface region mostly came from the cohesive force between the fiber and the matrix. The micrograph of the surface of the plain steel fiber (Figure 9b) was uneven due to NaCl corrosion, and there was a small amount of cement hydration product attached to the fiber due to the rougher surface. The content of Ca increased to 9.91%, Si increased to 4.41%, and Fe decreased to 76.53%.

It can be seen from the micrograph of the zinc phosphate modified steel fiber before and after the corrosion (Figure 9c,d) that, although there were microcracks on the surface of the phosphatized steel fiber after the corrosion, the overall surface morphology did not change significantly. The surface of steel fiber tended to be rough due to the existence of zinc phosphate coating. The surface of zinc phosphate modified steel fiber mainly contains O, Si, P, Ca, Fe, Zn elements (Table 5), indicating that the phosphating treatment of steel fiber has a great influence on the composition of elements in the interface area. In the samples without NaCl corrosion, the content of Ca element in zinc phosphate modified steel fiber increased by 385.35%, and the content of Fe element decreased by 80.21%, compared to the plain steel fiber. The same phenomenon can be found in the samples with NaCl corrosion: the content of Ca element in zinc phosphate modified steel fiber increased by 261.76%, and the content of Fe element decreased by 87.55%. The results show that the interface layer between phosphatized steel fiber and concrete matrix does not only contain conventional hydration products such as C–S–H and Ca(OH)_2_. Studies [14] have shown that phosphatized steel fiber can contribute to the formation of hydroxyapatite (Ca_5_(PO_4_)_3_(OH)) and calcium phosphate (CaHPO_4_·2H_2_O) in concrete. These products can fill the pores of the interface layer between the steel fiber and concrete matrix, which is also an important factor to improve the interface adhesion of the steel fiber and concrete matrix. In the mixing process of concrete, the passivation reaction does not occur on the steel fibers, but a more complex reaction takes place with water and concrete particles due to the presence of the zinc phosphate coating. This reduces the anisotropy between different materials, reduces the thickness of the interface layer, and improves the strength of the weakest point of the interface layer. When bearing the external load, the cracks can be prevented to some extent due to the increase in the bond strength of the interface. When cracks occur, the surface roughness of the phosphatized steel fiber can increase the work needed to pull out the fiber, achieving a toughening effect.

## 4. Conclusions

This paper provides a method to improve the chloride ion corrosion resistance of steel fiber reinforced concrete. Interfacial bond strength, micro-hardness, and micro-morphology properties of the concrete were respectively analyzed. The main findings are as follows:

(1) Phosphating treatment on steel fiber can increase the maximum load during the steel fiber pull-out of concrete in the presence of chloride ions. Furthermore, the bond strength between the steel fiber and concrete matrix is improved. The average bond strength of steel fiber reinforced concrete modified by zinc phosphate increased by 15.4% under the attack of chloride ion.
(2) Phosphating treatment decreases the thickness of the interface layer between steel fiber and concrete by 20 μm, as well as increasing its micro-hardness by around 20 HV, in both corroded and uncorroded environments. With regard to the plain steel fiber reinforced concrete, the presence of a corroded environment increases the thickness of the interface layer by 20 μm and decreases the micro-hardness by 15 HV, compared to that under an uncorroded environment. However, NaCl has little effect on the thickness and micro-hardness of the interface between steel fiber and concrete matrix in the steel fiber reinforced concrete modified by zinc phosphate.
(3) The overall surface morphology did not change much for the zinc phosphate modified steel fiber before and after corrosion, regardless of whether there were minor microcracks on the surface of the phosphatized steel fiber after corrosion.
(4) The steel fiber reinforced concrete modified by zinc phosphate shows good resistance to chloride ion corrosion.

## Figures and Tables

**Table 1:** Chemical composition and physical properties of Portland cement and fly ash.

Materials	CaO	SiO_2_	Al_2_O_3_	Fe_2_O_3_	MgO	SO_3_	R_2_O	Loss	d_mean_/μm	BET/(m^2^/g)
CEM 42.5	64.47	22.68	5.81	4.47	1.74	2.65	0.51	1.49	13.7	3.14
Fly ash	4.35	59.95	26.78	1.53	2.30	1.46	2	1.63	12.5	7.81

d_mean_ is average particle size. BET is specific suface area mesured by Brunner−Emmet−Teller method.

**Table 2:** The results of potentiodynamic corrosion tests.

No.	E_corr_ (mV)	I_corr_ (μA/cm^2^)	R_p_ × 10^3^ (Ω·cm^2^)	Corrosion Rate (g/m^2^h)	P (%)	Pe (%)
SF^a^	−805 ± 5.3	31.998 ± 1.2	3.06 ± 0.87	0.333	-	-
ZPP-SF^b^	−641 ± 5.2	8.721 ± 1.0	60.2 ± 3.2	0.091	1.53	72.7

SF^a^ is plain steel fiber. ZPP-SF^b^ is zinc phosphate modified steel fiber.

**Table 3:** Mixing proportions of the concrete.

Mixing Proportions of the Concrete (kg/m^3^)					Volume of Steel Fiber
Portland Cement	Fly Ash	Water	Sand	Gravel	
526	130	305	808	987	1%

**Table 4:** Bond strength of the interface between the steel fiber and concrete matrix.

Environment	Type of Fiber	Embedded Depth of the Steel Fiber/mm	Average Value of Maximum Pull-Out Load/N	Average Interfacial Bond Strength/MPa	Work Done When the Fiber Is Pulled Out/N·m
Corroded	SF	16	1218.56	6.35	0.60
	ZPP-SF	16	1407.81	7.33	0.69
Uncorroded	SF	16	1243.03	6.47	0.61
	ZPP-SF	16	1323.76	6.89	0.65

SF is plain steel fiber. ZPP-SF is zinc phosphate modified steel fiber.

**Table 5:** EDS of two kinds of steel fiber pulled out from the concrete before and after corrosion.

Types of Fiber	Environment	Elements						
		O	Al	Si	P	Ca	Fe	Zn
Plain	a	5.58	1.28	1.84	-	5.53	84.14	-
	b	8.54	0.61	4.41	-	9.91	76.53	-
Zinc phosphate treatment	a	22.65	2.43	7.5	5.86	26.84	16.65	17.07
	b	23.83	1.87	6.86	9.76	35.85	9.53	12.3

a Without corrosion, b NaCl solution.
